# Tumor necrosis factor-α decreases EC-SOD expression through DNA methylation

**DOI:** 10.3164/jcbn.16-111

**Published:** 2017-04-07

**Authors:** Shunpei Morisawa, Hiroyuki Yasuda, Tetsuro Kamiya, Hirokazu Hara, Tetsuo Adachi

**Affiliations:** 1 Laboratory of Clinical Pharmaceutics, Gifu Pharmaceutical University, 1-25-4 Daigaku-nishi, Gifu 501-1196, Japan

**Keywords:** extracellular-superoxide dismutase, tumor necrosis factor-α, epigenetics, DNA methylation

## Abstract

Extracellular-superoxide dismutase (EC-SOD) is a secreted antioxidative enzyme, and its presence in vascular walls may play an important role in protecting the vascular system against oxidative stress. EC-SOD expression in cultured cell lines is regulated by various cytokines including tumor necrosis factor-α (TNF-α). TNF-α is a major mediator of pathophysiological conditions and may induce or suppress the generation of various types of mediators. Epigenetics have been defined as mitotically heritable changes in gene expression that do not affect the DNA sequence, and include DNA methylation and histone modifications. The results of the present study demonstrated that TNF-α significantly decreased EC-SOD level in fibroblasts with an accompanying increase in methylated DNA. In DNA methylation and demethylation, cytosine is methylated to 5-methylcytosine (5mC) by DNA methyltransferase (DNMT), and 5mC is then converted to 5-hydroxymethylcytosine (5hmC) and cytosine in a stepwise manner by ten-eleven translocation methylcytosine dioxygenases (TETs). However, DNMT did not participate in TNF-α-induced DNA methylation within the *EC-SOD* promoter region. On the other hand, TNF-α significantly suppressed TET1 expression and EC-SOD mRNA levels were decreased by the silencing of TET1 in fibroblasts. These results demonstrate that the down-regulation of EC-SOD by TNF-α is regulated by DNA methylation through reductions in TET1.

## Introduction

Oxidative stress induces chronic inflammatory diseases such as atherosclerosis and diabetic complications, and is triggered by the excessive endogenous and exogenous production of reactive oxygen species (ROS) and/or their insufficient removal. Superoxide dismutase (SOD) is an antioxidative enzyme and its deficiency has been shown to increase the risk of various diseases.^(1–3)^ Extracellular-SOD (EC-SOD), one of the three SOD isozymes in mammals, is secreted into the extracellular space after its expression and is widely distributed in tissues.^(4)^ EC-SOD levels are high in the lung and kidney, and it is localized to specific cells in these tissues.^(5–7)^ However, it currently remains unclear why the expression of EC-SOD differs among cells and/or tissues. EC-SOD expression in cultured cell lines is regulated by various cytokines including tumor necrosis factor-α (TNF-α).^(8–10)^ TNF-α may induce or suppress the generation of various types of mediators, including cyclooxygenases, matrix metalloproteinases, and cytokines, resulting in the progression of inflammatory conditions.^(11,12)^ TNF-α is primarily secreted by inflammatory cells, and also by a broad variety of other cells including adipocytes.^(13,14)^ TNF-α inhibits insulin transduction, and disturbances in the metabolism of TNF-α have been implicated in metabolic disorders, such as obesity and insulin resistance indicating that perturbations in the metabolism of TNF-α affect the onset and progression of type 2 diabetes mellitus.^(15–17)^

Epigenetics have been defined as mitotically heritable changes in gene expression that do not change the DNA sequence, and include DNA methylation and histone modifications.^(18)^ DNA methylation, occurring at the 5' carbon of cytosine within CpG, plays a pivotal role in the regulation of tissue-specific gene expression.^(19)^ Regions with a high density of CpG sites are referred to as CpG islands, and are mostly unmethylated. However, gene expression is suppressed by the methylation of some CpG islands, and DNA methylation is involved in the development, differentiation, or termination of cells.^(20–22)^ DNA methylation is initiated by the transmethylation of *S*-adenosylmethionine, a methyl group donor, which is catalyzed by DNA methyltransferase (DNMT).^(23,24)^ On the other hand, methylated DNA is demethylated by ten-eleven translocation methylcytosine dioxygenases (TETs), which are characterized dioxygenases that catalyze the progressive oxidation of 5-methylcytosine (5mC) to produce 5-hydroxymethylcytosine (5hmC) and further oxidized derivatives.^(25)^ Recent studies demonstrated that the expression of human EC-SOD is regulated by DNA methylation and/or histone acetylation.^(26–29)^

EC-SOD activity was previously reported to be decreased in type 2 diabetes.^(30–32)^ Moreover, EC-SOD expression is suppressed by TNF-α.^(8–10)^ On the other hand, the enhanced expression of EC-SOD has been shown to mitigate streptozotocin-induced diabetic cardiomyopathy by attenuating oxidative stress.^(33)^ DNA methylation has been implicated in the weak expression of EC-SOD because DNA methylation within the *EC-SOD* promoter region inhibits the binding of the Sp1/3 transcriptional factor.^(27,34)^ TNF-α was recently shown to regulate gene expression by histone acetylation and methylation.^(35,36)^ In the present study, we investigated whether TNF-α down-regulates the expression of EC-SOD through the DNA methylation of its promoter region.

## Materials and Methods

### Cell culture

Normal human dermal fibroblasts were cultured in Dulbecco’s modified Eagle’s medium containing 10% (v/v) fetal calf serum, 100 units/ml penicillin, and 100 µg/ml streptomycin. Cells were maintained at 37°C in a humidified 5% CO_2_ incubator. Culture medium was replaced every 2 days.

### Real-time RT-PCR analysis

Fibroblasts (seeded at 4 × 10^5^ cells/dish on 60-mm culture dishes) were cultured overnight and then treated with TNF-α (R&D Systems, Minneapolis, MN) or 5-azacytidine (Aza, Wako Pure Chem. Ind., Osaka, Japan). After the treatment, cells were washed with cold phosphate-buffered saline (PBS) and total RNA was extracted from cells with TRIzol reagent (Invitrogen, Carlsbad, CA). cDNA was prepared by the method described in our previous study.^(37)^ Real-time RT-PCR was performed using Thunderbird^TM^ SYBR qPCR Mix (Toyobo, Osaka, Japan) according to the manufacturer’s protocol. The primer sequences used in real-time RT-PCR were shown in Table 1. mRNA levels were normalized to those of 18S rRNA mRNA in each sample.

### McrBC digestion of genomic DNA

Genomic DNA was isolated from fibroblasts using a Puregene Core kit (Qiagen, Chatsworth, CA) according to the manufacturer’s protocol. Genomic DNA was cleaved with EcoRI at 37°C for 2 h, followed by phenol-chloroform extraction and ethanol precipitation. Cleaved genomic DNA (500 ng) was further cleaved with McrBC, an endonuclease that cleaves DNA containing methylcytosine, in a final reaction volume of 10 µl at 37°C for 1 h, followed by an incubation at 65°C for 20 min. Cleaved genomic DNA was diluted twice with TE buffer (10 mM Tris-HCl pH 8.0, containing 1 mM EDTA), and 2-µl DNA samples were used as a template for the real-time RT-PCR analysis. The primer pairs used were as follows: sense 1 (−1,208 bp from the transcription start site) 5'-GCTGGTAACTAAGTCACCCA-3'; antisense 1 (−764 bp) 5'-TGTTGTCTGGGAGAACTAGG-3' and sense 2 (−729 bp) 5'-ATAACGGTAACGACAATGACAA-3'; antisense 2 (+51 bp) 5'-TAGCACCCACCTTTCCAGC-3'. After amplification, aliquots of the PCR mixtures were separated on a 2% (w/v) agarose gel, stained with ethidium bromide, and visualized using FLA5100 (Takara, Otsu, Japan).

### ELISA

EC-SOD concentrations in conditioned media of fibroblasts were measured by ELISA as described in our previous study.^(38)^

### Dot blot analysis

Genomic DNA was isolated from fibroblasts using a Puregene Core kit (Qiagen) according to the manufacturer’s protocol. Points were drawn by a pencil on Amersham Hybond-N^+^ (GE Healthcare, Tokyo, Japan) to indicate the region to be blotted. After the membrane was wetted, 3-µl DNA samples were spotted onto the membrane. After the membrane was dried, non-specific binding sites were blocked by soaking in 5% skimmed milk in PBS-T (PBS containing 0.03% Tween 20) in a container at room temperature for 1 h, and then washed 3 times with PBS-T. The membrane was incubated with an anti-5hmC antibody (1:1,000, Active motif, Carlsbad, CA) at 4°C overnight. After a washing step with PBS-T three times, the membrane was incubated with a horseradish peroxidase (HRP)-conjugated goat anti-rabbit antibody (1:3,000, Sigma-Aldrich, St. Louis, MO) at room temperature for 1 h. After washing 3 times with PBS-T, blots were detected using ImmunoStar LD (Wako Pure Chem. Ind.) and imaged using LAS-3000 UV mini (Fuji Film, Tokyo, Japan).

### Methylation-specific PCR analysis

Genomic DNA was isolated using a Puregene Core kit (Qiagen) according to the manufacturer’s protocol. The bisulfite modification of genomic DNA was performed using an EZ DNA Methylation-Gold kit (Zymo research, Irvine, CA) according to the manufacturer’s protocol. An aliquot of bisulfite-treated DNA (500 ng) was subjected to methylation-specific PCR (MSP) amplification. The primer sequences used in the MSP of the *EC-SOD* promoter were designed for the sodium bisulfite-modified template using MethPrimer software, and these MSP primer pairs were shown in Table 2. After amplification, aliquots of the PCR mixtures were separated on a 2% (w/v) agarose gel, stained with ethidium bromide, and visualized using FLA5100.

### Methylated DNA immunoprecipitation

Genomic DNA was isolated from fibroblasts using a Puregene Core kit (Qiagen) according to the manufacturer’s protocol. Genomic DNA (9 ng) was added to TE buffer to a total volume of 500 µl. Genomic DNA was sheared using the ultrasonic homogenizer Vivracell VC100 (Sonic & Materials, Danbury, CT) to achieve an estimated DNA size range of 150 to 800 bp and then boiled for 10 min. After the addition of 500 µl of IP buffer (20 mM Tris-HCl pH 8.0, containing 2 mM EDTA, 150 mM NaCl, and 1% Triton X-100), sheared genomic DNA was incubated with the anti-5hmC antibody overnight followed by an incubation with Dynabeads Protein G (Invitrogen) for 2 h. After the incubation, beads were sequentially washed with methylated DNA immunoprecipitation (MeDIP) washing buffer (20 mM Tris-HCl pH 8.0, containing 2 mM EDTA, 300 mM NaCl, 0.1% Triton X-100, and 0.1% SDS) and TE buffer, and then incubated in MeDIP elution buffer (25 mM Tris-HCl pH 8.0, containing 10 mM EDTA and 0.5% SDS) with proteinase K at 65°C for 2 h. After phenol-chloroform extraction and ethanol precipitation, genomic DNA was dissolved in 20 µl of TE buffer. The abundance of *EC-SOD* promoter regions in MeDIP precipitates was quantified using a PCR analysis. The primer sequences for EC-SOD were sense 5'-GTG GAGGCGAAGCAATTCTA-3'; antisense 5'-CTGTTAGCGCGA GTGCAGGA-3'. After amplification, these PCR products were loaded onto a 2% (w/v) agarose gel for electrophoresis and visualized using FLA5000, and a densitometric analysis of the PCR products was performed with Multi Gauge Ver. 3.0.

### Western blotting

The nuclear fraction and whole cell extract isolated from fibroblasts were boiled with sample buffer (62.5 mM Tris-HCl pH 6.8, containing 2% SDS, 10% glycerol, 50 mM dithiothreitol, and 0.01% bromophenol blue) for 5 min and then separated by SDS-PAGE on a 7.5% (w/v) polyacrylamide gel. This was followed by electrophoretic transferal onto PVDF membranes. Non-specific binding sites were blocked by soaking in 5% skimmed milk in PBS-T in a container at room temperature for 1 h and washing 3 times with PBS-T. The membranes were then incubated with the anti-TET1 antibody (1:1,000, Active motif) at 4°C overnight. After washing with PBS-T 3 times, the blots were incubated with the HRP-conjugated goat anti-rabbit antibody (1:3,000) for 1 h. Bands were detected using Super-Signal West Pico (Thermo Scientific, Rockford, IL) and imaged using LAS-3000 UV mini (Fuji Film).

### Small interfering TET1 transfection

Fibroblasts were grown on 60-mm dishes and transiently transfected with TET1-specific small interfering RNA (siRNA). The siRNA of TET1 (#147894) and negative control siRNAs were purchased from Thermo Scientific. Lipofectamine^TM^ RNAiMAX Transfection Reagent (Invitrogen) was used for siRNA transfection according to the manufacture’s protocol.

### Data analysis

Data are expressed as the means ± SD of three independent experiments. Statistical evaluations of the data obtained were performed using ANOVA followed by post-hoc Bonferroni tests. A *p* value less than 0.05 was considered significant.

## Results

### Effects of TNF-α on EC-SOD expression in fibroblasts

**Fig. 1:**
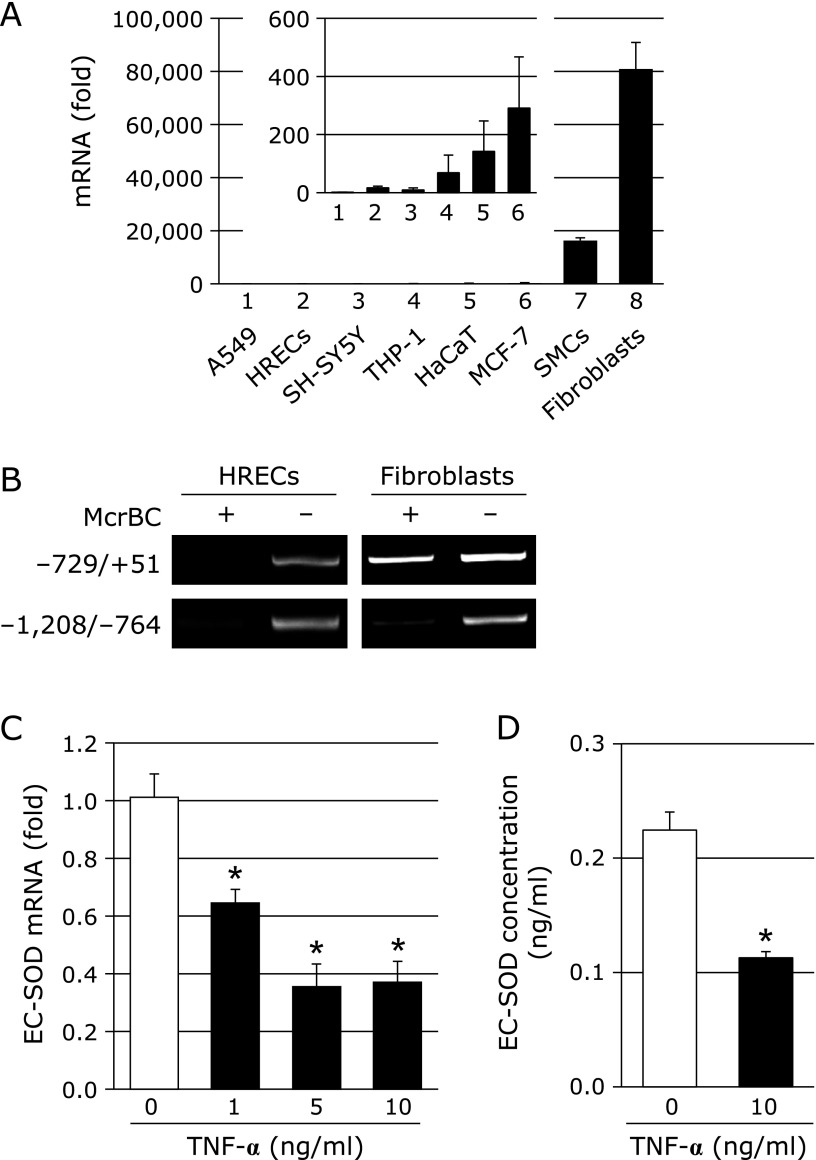
(A) EC-SOD mRNA levels in various cells. (1) A549, human lung adenocarcinoma epithelial cells; (2) HRECs, human retinal endothelial cells; (3) SH-SY5Y, human neuroblastoma; (4) THP-1, human monocytic leukemia cell line; (5) HaCaT, human skin keratinocytes; (6) MCF-7, human breast cancer cell line; (7) SMCs, human vascular smooth muscle cells; (8) fibroblasts, normal human dermal fibroblasts. Inserted figure shows the EC-SOD mRNA levels in cell Nos. (1) to (6). EC-SOD mRNA levels in cell Nos. (2) to (8) were compared to those in A549 cells (1). (B) Methylation status of the *EC-SOD* promoter. McrBC digestion analyses were performed on fibroblasts and HRECs. (C) Effects of TNF-α on EC-SOD expression in fibroblasts. Fibroblasts were treated with or without TNF-α (1–10 ng/ml) for 72 h, and EC-SOD mRNA levels were measured by real-time RT-PCR. Real-time RT-PCR data were normalized using 18S rRNA. *p<0.01 vs untreated cells. (D) Fibroblasts were treated with TNF-α (10 ng/ml) for 72 h. After the treatment, EC-SOD levels were measured by ELISA. *p<0.01 vs untreated cells.

EC-SOD is known to be strongly expressed in fibroblasts.^(39)^ We also found that the expression levels of EC-SOD in fibroblasts were markedly higher than those in other culture cells, as shown in Fig. 1A. EC-SOD expression was previously reported to be regulated by DNA methylation within the *EC-SOD* promoter region.^(27)^ We showed that the CpG site at *EC-SOD* promoter regions −452 to −207 in fibroblasts was unmethylated, whereas it was methylated in human retinal endothelial cells (HRECs), which weakly express EC-SOD.^(40)^ A PCR analysis using McrBC showed that the *EC-SOD *promoter region from –729 to +51 in HRECs was digested by McrBC, while amplification in fibroblasts was resistant to McrBC (Fig. 1B). These results suggest that the strong expression of EC-SOD in fibroblasts is involved in DNA demethylation in a narrow range of CpG sites in the *EC-SOD* promoter region.

TNF-α has been suggested to regulate the expression of many genes. We confirmed that the treatment of fibroblasts with TNF-α significantly decreased EC-SOD mRNA levels in dose-dependent (Fig. 1C) and time-dependent manners (data not shown) under our experimental conditions. Moreover, EC-SOD protein levels were decreased by the treatment with TNF-α (Fig. 1D).

### Effects of TNF-α on DNA methylation and hydroxymethylation within the *EC-SOD* promoter region

**Fig. 2:**
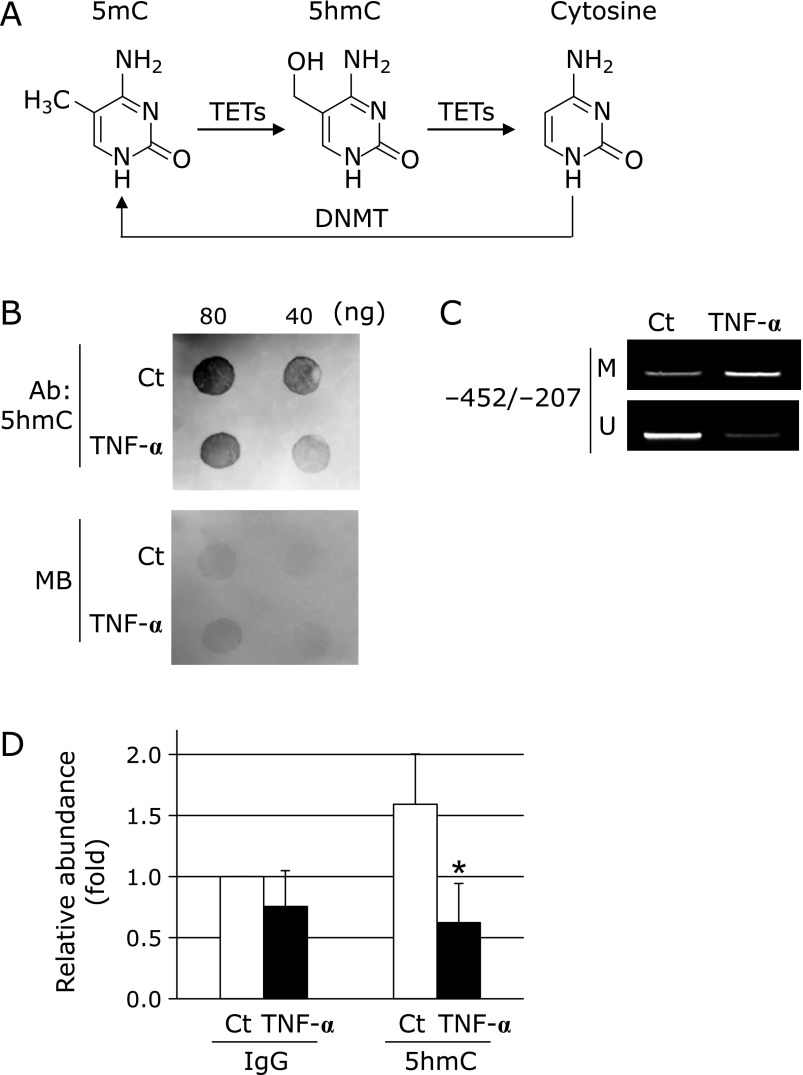
Involvement of DNA methylation in TNF-α-inducible EC-SOD down-regulation in fibroblasts. (A) DNA methylation of cytosine by DNMT and demethylation of 5mC by TETs. (B) Down-regulation of 5hmC by TNF-α. Fibroblasts were treated with or without 10 ng/ml TNF-α for 72 h. Isolated genomic DNA (80 or 40 ng) was subjected to a DNA dot blot analysis with an anti-5hmC antibody. Protein concentrations in samples were confirmed by staining with methylene blue (MB). (C) Fibroblasts were treated with or without 10 ng/ml TNF-α for 72 h, followed by bisulfite-MSP amplification with methylation (M) and unmethylation (U) site primers. (D) Change in 5hmC on the *EC-SOD* promoter by TNF-α. Fibroblasts were treated with or without 10 ng/ml TNF-α for 72 h, followed by a MeDIP analysis with a 5hmC antibody. *p<0.05 vs control (Ct).

In DNA methylation and demethylation, cytosine is methylated to 5mC by DNMT, and 5mC is then sequentially converted to 5hmC and cytosine by TETs (Fig. 2A). As shown in Fig. 2B, the treatment of fibroblasts with TNF-α decreased global 5hmC. Furthermore, we investigated whether TNF-α changes the levels of DNA methylation and demethylation at the *EC-SOD* promoter region using an MSP analysis and MeDIP, respectively. DNA methylation within the *EC-SOD* promoter region from −452 to −207 was increased by the treatment with TNF-α (Fig. 2C), while DNA hydroxymethylation within the *EC-SOD* proximal promoter region was significantly decreased (Fig. 2D). These results suggest that the TNF-α-induced down-regulation of EC-SOD is involved in increases in DNA methylation and/or decreases in hydroxymethylation.

### Involvement of TETs in TNF-α-induced down-regulation of EC-SOD

**Fig. 3:**
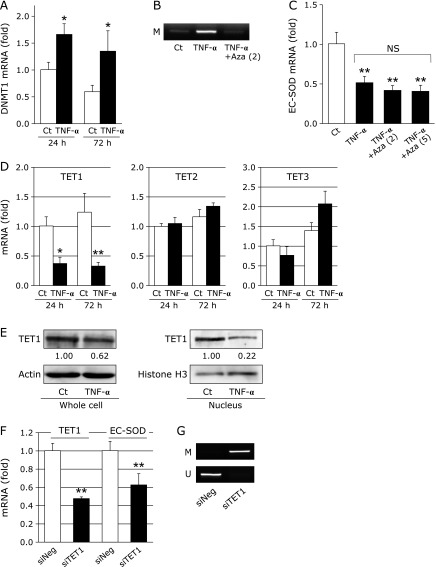
Involvement of DNMT and TETs in EC-SOD expression. (A) Fibroblasts were treated with or without TNF-α (10 ng/ml) for 24 or 72 h, followed by the measurement of DNMT1 mRNA levels by real-time RT-PCR. Real-time RT-PCR data were normalized using 18S rRNA. *p<0.05 vs control (Ct). (B) Fibroblasts were treated with or without 10 ng/ml TNF-α and 2 µM Aza for 72 h, followed by bisulfite-MSP amplification with methylation site primers (–452/–207). (C) Fibroblasts were treated with or without 10 ng/ml TNF-α and Aza (2 or 5 µM) for 72 h, followed by the measurement of EC-SOD mRNA levels by real-time RT-PCR. Real-time RT-PCR data were normalized using 18S rRNA. **p<0.01 vs control (Ct); NS, not significant. (D) Fibroblasts were treated with or without TNF-α (10 ng/ml) for 24 or 72 h, followed by the measurement of TET mRNA levels by real-time RT-PCR. Real-time RT-PCR data were normalized using 18S rRNA. *p<0.05, **p<0.01 vs control (Ct). (E) Fibroblasts were treated with or without TNF-α (10 ng/ml) for 72 h, and TET1 protein levels in whole cell extracts or nuclear fractions were then measured by Western blotting. Values are fold changes from the control. (F) Fibroblasts were treated with TET1 siRNA (siTET1) or negative control siRNA (siNeg) for 24 h, followed by a cell culture for 48 h. EC-SOD and TET1 mRNA levels were then measured by real-time RT-PCR. Real-time RT-PCR data were normalized using 18S rRNA. **p<0.01 vs siNeg. (G) Fibroblasts were treated with siTET1 or siNeg for 24 h, followed by a cell culture for 48 h and then bisulfite-MSP amplification with methylation (M) and unmethylation (U) site primers (–452/–207).

DNA methylation is known to be mediated by DNMT.^(41)^ The treatment with TNF-α for 24 or 72 h significantly increased DNMT1 mRNA levels, as shown in Fig. 3A. The addition of Aza, a DNMT inhibitor, suppressed DNA methylation induced by TNF-α at the *EC-SOD* promoter region (Fig. 3B). However, suppression of EC-SOD by the treatment with TNF-α was not attenuated by the addition of Aza (Fig. 3C).

TETs are recently characterized dioxygenases that catalyze the progressive oxidation of 5mC to produce 5hmC and further oxidized derivatives.^(41)^ The treatment with TNF-α for 24 h or 72 h significantly decreased TET1 mRNA levels, but did not affect TET2 or TET3 mRNA levels (Fig. 3D). Moreover, TET1 protein levels in whole cells and nuclear fractions were decreased by the treatment with TNF-α for 72 h (Fig. 3E). We investigated whether EC-SOD expression correlates with TET1 expression. As shown in Fig. 3F, EC-SOD mRNA levels were significantly decreased by the silencing of TET1 in fibroblasts. DNA methylation at the *EC-SOD* promoter region was increased by the silencing of TET1 (Fig. 3G). These results suggest that TNF-α suppresses EC-SOD expression induced by the down-regulation of TET1.

## Discussion

EC-SOD mainly defends against extracellular ROS, which have been implicated in diabetes, arteriosclerosis, and other diseases. The treatment of cardiovascular tissues with EC-SOD reduces the extent of damage and improves cardiac function, for example, reductions in the remodeling of vasculature, the attenuation of apoptosis, inhibition of smooth muscle cell migration, and recovery of the endothelial cell layer.^(42)^ On the other hand, reductions in EC-SOD have been shown to induce severe IL-23-mediated skin inflammation in mice.^(43)^ A previous study reported that EC-SOD protein levels were lower in skin tissues isolated from type 2 diabetes patients than in normal tissues.^(44)^ Several genes involved in these diseases, including the *EC-SOD* gene, are regulated by epigenetics. DNA methylation in the promoter region is known to suppress gene transcription. We recently demonstrated that the expression of EC-SOD in monocytes also regulated DNA methylation and/or histone acetylation.^(28)^ The results of the present study showed that the CpG site within the *EC-SOD* promoter region in fibroblasts is unmethylated and this state induces the strong transcriptional activity of the *EC-SOD* gene.

TNF-α induces the generation of various types of mediators, and the expression levels of TNF-α are known to be high in inflammatory diseases and diabetes. We previously reported that the treatment with pioglitazone, an anti-diabetic agent, increased plasma EC-SOD and adiponectin levels and decreased TNF-α levels.^(31)^ Fig. 1C and D show that EC-SOD mRNA and protein levels in fibroblasts were decreased by the treatment with TNF-α. TNF-α regulates gene expression by histone modifications and DNA methylation.^(35,36,45)^ In the classical signaling pathway stimulated by TNF-α, an upstream IκB kinase complex (IKK) is activated that results in the phosphorylation and degradation of IκB proteins, releasing the nuclear factor-κB (NF-κB) dimer, which, in turn, translocates to the nucleus and activates transcription.^(46,47)^ As shown in Fig. 2C, the treatment with TNF-α increased methylated CpG sites within the *EC-SOD* promoter region, suggesting the possibility that the reductions induced in EC-SOD expression by the treatment with TNF-α were caused by DNA methylation within the *EC-SOD* promoter region. The methylation of cytosine is mediated by DNMT. TNF-α has been reported to increase DNMT1 expression and promote DNMT3B recruitment on the gene promoter.^(45,48)^ We also observed the increase of DNMT1 mRNA by TNF-α treatment. However, EC-SOD mRNA levels were not changed by the addition of Aza, whereas Aza attenuated the DNA methylation induced by the treatment with TNF-α, as shown in Fig. 3A–C. These results indicate that TNF-α-induced DNA methylation within this *EC-SOD* promoter region may not be due to DNMT activation.

TETs were recently found to play a role in the mechanism responsible for DNA demethylation.^(41)^ Furthermore, TET1 has been shown to inhibit gastric cancer growth and metastasis,^(49)^ and TET activity is decreased by excessive glucose.^(50)^ The results shown in Fig. 2 and 3 revealed that 5hmC levels and TET1 expression were decreased by the treatment with TNF-α. We recently discovered that the expression of EC-SOD was increased by the transient overexpression of TET1 in A549 cells (unpublished data). In the present study, we evaluated the contribution of TET1 to EC-SOD expression by depleting TET1 using TET1 siRNA. As shown in Fig. 3F and G, EC-SOD expression was significantly decreased by TET1 silencing in fibroblasts accompanied with the increase of DNA methylation at the *EC-SOD* promoter region. These results suggest that the strong expression of EC-SOD in fibroblasts depends on the increases of DNA demethylation within the EC-SOD promoter region by TET1 expression. It is possible that activation of NF-κB signaling by TNF-α down-regulates EC-SOD through negatively regulation of TET1 expression, because the down-regulation of TET1 was inhibited by NF-κB inhibitors in chondrocytes.^(51)^

EC-SOD expression correlates with various diseases and is regulated by TNF-α. TNF-α expression influences various diseases, and we speculate that TNF-α may play a pivotal role in the down-regulation of TET expression, resulting in disease progression. The results of the present study suggest that the down-regulation of EC-SOD by TNF-α is regulated by DNA methylation through reductions in TET1.

## Figures and Tables

**Table 1:** Primer sequences used in real-time RT-PCR

Primer		Sequences
EC-SOD	forward	5'-AGAAAGCTCTCTTGGAGGAG-3'
	reverse	5'-TACATGTCTCGGATCCACTC-3'
DNMT1	forward	5'-ACCGCTTCTACTTCCTCGAGGCCTA-3'
	reverse	5'-GTTGCAGTCCTCTGTGAACACTGTGG-3'
TET1	forward	5'-TCTGTTGTTGTGCCTCTGGA-3'
	reverse	5'-CCCATGACCACATCTACTGT-3'
TET2	forward	5'-AGCAATAGGACATCCCTGAG-3'
	reverse	5'-CATCTAGGAGCAGGTCCTAA-3'
TET3	forward	5'-CGGATCGAGAAGGTCATCTA-3'
	reverse	5'-ATGACGATCACAGCGTTCTG-3'
18S rRNA	forward	5'-CGGCTACCACATCCAAGGAA-3'
	reverse	5'-GCTGGAATTACCGCGGCT-3'

**Table 2:** Primer sequences used in the MSP analysis

Primer		Sequences
–452/–207 — (M)	forward	5'-TATAGTTTTGGAGTAAATGTTACGT-3'
	reverse	5'-CTCCCATTTTTAAATTTTCGAA-3'
–452/–207 — (U)	forward	5'-TAGTTTTGGAGTAAATGTTATGT-3'
	reverse	5'-CCTCCCATTTTTAAATTTTCAAA-3'

## Abbreviations

Aza: 5-azacytidine
DNMT: DNA methyltransferase
EC-SOD: extracellular-superoxide dismutase
5hmc: 5-hydroxymethylcytosine
HRECs: human retinal endothelial cells
HRP: horseradish peroxidase
5mC: 5-methylcytosine
MeDIP: methylated DNA immunoprecipitation
MSP: methylation-specific polymerase chain reaction
ROS: reactive oxygen species
siRNA: small interfering RNA
TET: ten-eleven translocation methylcytosine dioxygenase
TNF-α: tumor necrosis factor-α
